# Rhythmic Chanting and Mystical States across Traditions

**DOI:** 10.3390/brainsci11010101

**Published:** 2021-01-13

**Authors:** Gemma Perry, Vince Polito, William Forde Thompson

**Affiliations:** 1 Department of Psychology, Macquarie University, Sydney 2109, Australia; gemma.perry@hdr.mq.edu.au; 2 Department of Cognitive Science, Macquarie University, Sydney 2109, Australia; vince.polito@mq.edu.au; 3 ARC Centre of Excellence in Cognition and Its Disorders, Department of Psychology, Macquarie University, Sydney 2109, Australia

**Keywords:** chanting, music, cross cultural, mystical experience, altered state, meditation, mantra, religiosity, absorption, altruism

## Abstract

Chanting is a form of rhythmic, repetitive vocalization practiced in a wide range of cultures. It is used in spiritual practice to strengthen community, heal illness, and overcome psychological and emotional difficulties. In many traditions, chanting is used to induce mystical states, an altered state of consciousness characterised by a profound sense of peace. Despite the global prevalence of chanting, its psychological effects are poorly understood. This investigation examined the psychological and contextual factors associated with mystical states during chanting. Data were analyzed from 464 participants across 33 countries who regularly engaged in chanting. Results showed that 60% of participants experienced mystical states during chanting. Absorption, altruism, and religiosity were higher among people who reported mystical states while chanting compared to those who did not report mystical states. There was no difference in mystical experience scores between vocal, silent, group or individual chanting and no difference in the prevalence of mystical states across chanting traditions. However, an analysis of subscales suggested that mystical experiences were especially characterised by positive mood and feelings of ineffability. The research sheds new light on factors that impact upon chanting experiences. A framework for understanding mystical states during chanting is proposed.

## 1. Introduction

Chanting involves rhythmic, repetitive singing or speaking of sounds and phrases either vocally or mentally [1]. It is practiced globally in many different cultures and for a multitude of functions such as strengthening communities, healing illness, or overcoming psychological and emotional difficulties [2,3]. Although chanting rituals take various forms, they are often used to induce altered states of consciousness [4]. Shamanic traditions use chanting in order to enter altered states believed to be vital for healing and modifying behaviour [5]; Indigenous Australians use chanting to connect with the Ancestral world [6]; Sufism uses chanting, dance and breathing to connect to God and enter into states of trance [7]; and Yogic traditions believe that chanting practices are a way of reaching states of consciousness beyond the disturbances of the mind [8].

Altered states of consciousness associated with rituals such as chanting can take many forms that range from the relatively mundane, such as being absorbed in a task, to intense experiences of complete loss of individual identity [9,10,11]. Here, we focus on mystical experience, an altered state of consciousness characterised by a profound sense of peace and feelings of unity with objects or persons perceived in one’s surroundings [12,13]. Mystical experiences often involve overwhelming positive emotions, distortion of time and space, and a loss of boundaries between oneself, others and nature [4,14,15]. These experiences can be sustained for hours, days, or weeks, and sometimes include a complete revelation that permanently transforms how an individual behaves in the world [16]. Such states are frequently reported as being the most meaningful event in someone’s life and are often associated with positive outcomes such as prosocial behaviour, increased self-awareness, health and well-being [4,17]. Therefore, chanting rituals may go beyond the widely reported psychosocial benefits of other music practices [18,19] and trigger significant transformation in an individual’s life.

Despite the worldwide prevalence of chanting and its apparent transformative effects, there is surprisingly little empirical research on its psychosocial impact. Preliminary evidence suggests that chanting practices lead to decreased anxiety and depression, and increased positive mood, focused attention and relaxation [20,21,22]. However, important questions remain. How reliably does chanting induce altered states of consciousness? What are the characteristics of altered states of consciousness induced by chanting? What are the mechanisms underlying these changes? It is unknown whether certain traditions or techniques have different psychological effects, or whether certain individual traits and belief systems enhance the experiential impact of chanting. The goal of this investigation was to examine how different characteristics and beliefs associated with chanting relate to psychological outcomes and altered states of consciousness such as mystical states.

Although research suggests that altered states can have important medical and psychological benefits, most of this research has focused on the use of serotonergic psychedelics [4,17]. It is therefore important to investigate other means of obtaining altered states that avoid the risks associated with psychedelics. In a survey performed on mystical states, 75% of participants reported their mystical experience as being one of the most spiritually significant events in their life regardless of whether the altered state was pharmacologically induced or not [14]. The same participants also reported that the experience had moderate to strong persisting positive changes to their mental health.

The goal of chanting in many traditions is to promote healing, social connection, and go beyond mundane states of awareness by altering states of consciousness [17,23,24]. There are two broad aspects of chanting that may contribute to promoting these states. Psychological processes could be triggered by (1) the mechanics of chanting, such as rhythm, repetition, focused attention, and synchrony; (2) the deep contemplation of the meaning, intention, or belief systems of the sounds being chanted. These two broad aspects of chanting may contribute to various outcomes of the practice, individually and jointly, as both have been found to have effects on the psyche independently [25].

There is a body of literature on altered states of consciousness [4,17,26,27], but the unique connection between chanting practices and such states is still poorly understood. One way of inducing an altered state is by increasing the intensity and variability of sensory input [11,28]. Accordingly, effortful chanting practices with complex rhythmic patterns may be particularly adapted to consciousness change. For example, Taketina, a practice described as an “active rhythm meditation,” developed by composer Reinhard Flescher, requires that participants maintain three separate rhythms while chanting, clapping, and stepping. Sufism also features rhythmic movements, where repetitive actions such as whirling and dancing accompany chanting [29]. Such complex movements are known to produce anxiolytic effects by stimulating the endocannabinoid system [30]. Combining rhythmic entrainment with other physiological effects of chanting might promote altered states to a greater extent than simple chanting practices such as repeating the syllable om, as used in Buddhist, Hindu and Yogic traditions [31].

Another possibility is that strong belief systems interact with rhythmic attending and vocalizing to produce altered states of consciousness. In that case, Taketina would not necessarily induce such states, because belief systems are not central to this practice. Other traditions, however, are associated with powerful belief systems surrounding a universal ‘oneness’ and could promote altered states through the use of these ritualistic intentions [25,32].

Chanting traditions are often imbued in a set of traditions or belief systems. For example, Indigenous Australians chant about how mythical creatures formed the land [6], Hindus chant to worship deities [33], and Muslims chant as a way of surrendering to God and showing their devotion [34]. Strong belief systems are not merely comforting; they can enhance people’s ability to recover and heal from trauma or physical injury [35]. The prevalence of strong belief systems in most chanting practices implies that such beliefs may play an important role in altered states of consciousness, implicating the possible involvement of brain regions and changes associated with religion and spirituality [12].

Despite variability in chanting traditions and associated beliefs, certain characteristics are common across chanting practices. Chanting can be performed silently or vocally, and individually or in a group, but most chanting practices involve the use of a predictable, repetitive rhythm, focused attention and, if practiced in groups, synchrony [36]. Both the unique and shared features of chanting traditions may be relevant to the psychosocial benefits of chanting. For example, silent individual chanting requires a high level of focus and discipline. Conversely, vocal group chanting has the additional features of coordinated vocalization, breath control and interpersonal synchrony which may strengthen social connections and community identity.

Interpersonal synchrony, one of the features involved in chanting, may also mediate the effect of chanting on mystical states. Chanting in a group promotes social connection and cooperation through synchronized rhythmic vocalization and movements. These coordinated temporal predictions have been found to increase social connection and promote oscillatory entrainment, in which brain rhythms and external rhythms synchronize [37,38,39,40,41]. Further, rhythmic coordination with others requires divided attention, which is cognitively demanding. Participants must integrate perceptual feedback from multiple sources simultaneously, attending both to their own vocalizations and movements and to those of others [41]. In the context of chanting, not only is an individual synchronizing to the chanting but is also interacting to the motor responses of others via facial expression, hand gestures, clapping, dancing, or swaying. Perception–action coupling promotes social interaction by combining a cognitively demanding task with high sensory engagement. This rhythmic social interaction often promotes the sense of merging with others and the music, which is a common experience of altered states of consciousness [17,42]

Chanting may promote social connection, leading to mystical states. However, mystical states might also promote social connection. After-effects of altered states of consciousness often include a more positive view of self and others, with a large majority of participants reporting that the experiences had improved their relationships with others [16]. Consequently, mystical experiences may also promote social cohesion as they are often accompanied by a loss of boundaries between self and others, resulting in strong feelings of connection to others, often described as a reduction in self-centeredness [4].

Many religions also promote pro-social behaviour by prioritizing others above the self [43]. For example, in Buddhist traditions, there is a large focus on qualities such as compassion and loving kindness [44]. Therefore, as well as chanting sounds, individuals may also have intentions during the chanting practice to act compassionately and kindly to others, or to focus attention on the ‘oneness of the universe’, reinforcing community and connection to others. These intentions may enhance the experience of chanting as well as nurturing altered states of consciousness.

Abraham Maslow was one of the first to investigate and define an altered state of consciousness referred to as a ‘peak experience’ [45]. He noted peak experiences can often occur during music listening, because music triggers a number of mechanisms that induce significant changes in mood, arousal and emotional experience [46]. Chanting may operate in a similar way, but with a strong emphasis on focused attention, synchronization, and often overlaid with powerful belief systems. This combination of psychological elements may be ideally suited for promoting well-being, social connection and altered states of consciousness [47]. Shared predictable, repetitive rhythms allow interpersonal synchrony between people and may contribute to the release of neurohormones involved in social connection [48]. Shifts in tempo are also used strategically in chanting practices to nurture feelings of bliss or ecstasy that may be accompanied by dancing and clapping. In kirtan, a style of call and response chanting originating in India, there is often a progression of the music that begins with a slow tempo, increases to a peak tempo that may be double or triple the original tempo, and then gradually returns to a meditative pace towards the end of the chant. Temporal repetition has been found to shift attention from local levels of music organisation in the brain to more global levels of organisation such that the music is ‘felt rather than thought’ [48].

**Figure 1:**
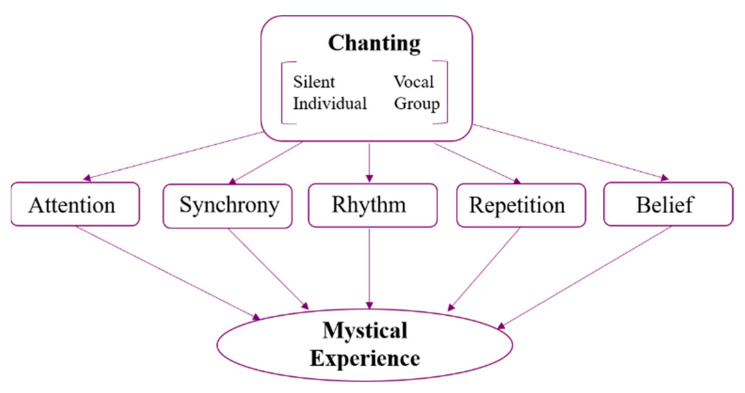
Convergence of features that may promote mystical experience during chanting.

Figure 1 depicts the most common characteristics of chanting, illustrating how structural features of music, belief systems, focused attention, and behavioural aspects of chanting converge to induce mystical states.

Little is known about skills or personality traits associated with mystical states. Absorption—the extent to which someone tends to become immersed in an experience—may be an important component of mystical and other altered states of consciousness [49]. As some people are more prone than others to absorption, it is considered a disposition or personality trait. Individuals that are high in absorption may experience total attentional engagement in an activity providing an immersion in the experience [50]. Absorption also includes the openness to being deeply affected by art or music and has been found to be associated with feelings of self-transcendence and distortions in time perception [50,51,52,53,54]. Taken together, these findings suggest that high levels of absorption may contribute to mystical states during chanting practices.

The current study set out to determine the prevalence and nature of mystical experiences in a wide range of chanting practices. An online survey was used to determine whether specific traits (absorption, altruism, religiosity) are associated with mystical experiences during chanting. The survey also examined whether types of practices (vocal, silent, group, individual) are associated with mystical experiences. Lastly, this study investigated whether mystical experiences differed across chanting practices and traditions.

In view of existing theory and evidence, several predictions were made. Firstly, absorption, altruism, and religiosity should be higher among participants who report mystical experiences compared to participants who do not report these experiences while chanting. Secondly, because vocal chanting allows for synchronization and direct physiological effects, it should be associated with mystical states more than silent chanting. Thirdly, group chanting provides a more complex process of rhythmic synchronization than individual chanting and therefore is more likely to be associated with mystical states. Although there is insufficient literature to make clear predictions about differences between chanting traditions, those associated with strong belief systems or complex rhythmic patterns should give rise to mystical states.

## 2. Materials and Methods

### 2.1. Participants

#### 2.1.1. Recruitment

Participants who were engaged in regular chanting practices, between the age of 18 and 80 years, and proficient in English were recruited via social media platforms, community newsletters and notice boards. There were two methods of recruitment: the survey was distributed through contacts embedded in chanting communities and posted in online forums connected to chanting groups. Participants were offered the chance to go into a prize draw to win one of five AUD$100 Amazon vouchers. Ethics approval was given by the Macquarie University Ethics committee and each participant provided electronic consent.

#### 2.1.2. Survey Responses

A total of 707 people opened the survey, and 456 completed it. An additional 8 participants completed at least 85% of the survey and all measures examined in the current investigation, so were also included in the analyses. All 464 participants in the final sample had engaged in chanting at least twice in the last month, so met our inclusion criteria.

Ages in the sample ranged from 18 to 78 years (*M* = 48.28, *SD* = 12.67). Participants were 75.4% female and 24.6% male. Chanters from 33 countries around the world participated in the survey. The most represented countries included Australia (45.7%), the United States (26.9%), the United Kingdom (5%), Canada (4.7%), India (3.2%), Germany (2.4%), the Netherlands (1.7%), Austria (1.7%), and New Zealand (1.1%), with the remainder (7.6%) from countries with less than 1% of participants.

### 2.2. Materials

Advertisements were posted on social media, chanting community notice boards and newsletters with a link to the online survey. After providing consent, participants first answered demographic questions and reported their style of chanting practice, before completing a series of psychometric scales, which were presented in counterbalanced order. Results from the following scales will be reported. Other measures were obtained but concerned different issues so will be reported in a separate manuscript.

#### 2.2.1. The Modified Tellegen Absorption Scale

Absorption was measured with The Modified Tellegen Absorption Scale (MODTAS) [55] which is a 34-item measure with a 5-point scale ranging from 0 (never) to 4 (very often). Example items include “when listening to organ music or other powerful music, I feel as if I am being lifted into the air” and “I am deeply moved by a sunset” and “I feel as though my mind could envelope the whole world.” Total scores range from 0 to 136 with higher scores reflecting higher absorption. The MODTAS has been reported to have an internal reliability of α = 0.96 [55,56].

#### 2.2.2. The Adapted Self-Report Altruism Scale

Altruism was measured with The Adapted Self-Report Altruism Scale (SRA) [57] which is a 14-item measure where participants rate the frequency that they engage in various altruistic behaviours. Each item is rated on a 5-point scale ranging from 1 (never) to 5 (very often). Example items include “I would give money to a charity” and “I would offer my seat on a train or bus to someone who was standing.” Total scores range from 14 to 70 with higher scores indicating more altruistic behaviour. This scale has been assessed by comparing it with an omnibus personality inventory and discriminant validity has been found to be good [58]. Further, the correlation between social desirability and the SRA (*r* = 0.05) indicates that scores on this measure are not influenced by socially desirable responding.

#### 2.2.3. The Centrality of Religiosity Scale

Religiosity was measured using The Centrality of Religiosity Scale (CRS) [33], which is a 15-item measure with the option of an additional 5 items for research across multiple religions (used here), consisting of questions regarding people’s religiosity. When using the CRS in surveys with Buddhists, Muslims and Hindus (as in the current study), it is recommended to use phrasing that reflects openness for polytheistic and other spiritual practices. This includes using the phrase “God, deities or something divine” rather than simply “God or something divine” in questions 2, 5, 10 and 15 [33]. There are 8 questions on the importance of religion which are rated on a 5-point scale ranging from 1 (never) to 5 (very often). Example statements include “How often do you experience situations in which you have the feeling that you are touched by a divine power?” and “How often do you pray spontaneously when inspired by daily situations?” There are 12 questions on the frequency of certain religious behaviours which are rated on a 5-point scale ranging from 1 (not at all) to 5 (very much so). Example statements include “How important is meditation for you?” and “To what extent do you believe that God, deities or something divine exists?” Scores range from 20 to 100 with higher scores reflecting higher religiosity or stronger religious beliefs. The 15-item CRS is the version that allows for highest reliability and accuracy ranging from 0.92 to 0.96. Validity of the CRS has also been confirmed through high correlations of religious identity and the CRS ranging from 0.73 to 0.83 [33].

#### 2.2.4. The Revised Mystical Experience Questionnaire

Altered states of consciousness in the context of chanting were measured using the revised mystical experience questionnaire (MEQ30) [59] which is a 30-item measure constructed from the MEQ43 [26] and measures complete mystical experiences as an extreme altered state of consciousness. It includes 4 factors: mystical, positive mood, transcendence of time and space, and ineffability. Each statement is rated on a 6-point scale ranging from 0 (none, not at all) to 5 (extreme). Example items include “Experience of oneness or unity with objects and/or persons perceived in your surroundings.” and “Feeling that you experienced something profoundly sacred and holy.” Scores range from 0 to 150, with higher scores reflecting higher mystical experiences. The criterion for a ‘complete mystical state’ is a score over 60% on each of the 4 factors [59]. The MEQ30 has been found to show excellent reliability and validity as well as good reliability for the factor subscales [26,59]. It has also been found to be robust to differences in sample demographics such as gender and education level [59]. 

## 3. Results

### 3.1. Descriptive Statistics

#### 3.1.1. Chanting Traditions

Participants were asked to indicate what tradition of chanting they practice most. The final sample included practitioners of Vedic chanting (23.1%), Hindu (14%), Buddhist (10.3%), Yoga (Kundalini Yoga, Satyananda Yoga, Sivananda Yoga; 9.7%), Hare Krishna (6.9%), Taketina (5.8%), Tantra (5.6%), Transcendental Meditation (Transcendental Meditation (TM), Primordial Sound Meditation, Vedic Meditation; 5.6%), Kirtan (Kirtan, Bhakti; 5.4%), and other (13.6%). Participants who did not indicate a category or indicated a chanting tradition with fewer than 20 participants were included in the ‘other’ category. Although participants were obliged to nominate just one chanting practice, it should be noted that many of these chanting traditions are not mutually exclusive, and have overlapping practices, themes and complex intertwined histories.

#### 3.1.2. Chanting Experience

Participants reported to have been chanting from 1 to 725 months (*M* = 129.03, *SD* = 134.627). When asked how regularly they had been chanting in the last 12 months, 62.7% reported chanting once or more every day, 24.1% once per week, 9.5% one to two times per month, and 3.7% less than once per month. When asked about the length of each chanting session, 53.7% reported chanting between ten minutes and one hour, 25.4% one hour or more and 20.9% ten minutes or less.

#### 3.1.3. Chanting Contexts

When asked what method of chanting they most often engaged in, most participants reported ‘call and response’ chanting (33%), followed by repetitive prayer (31.3%), repetitive prayer with beads (25.9%) and other (9.9%). More participants reported to be chanting vocally (75.9%) than silently (24.1%). Most participants reported chanting individually (67.5%) compared with chanting in groups (32.5%). Finally, most participants reported chanting at home (67.2%), with less reporting chanting in a temple, church or yoga studio (18.8%), at social events (3.2%), at work (0.9%), and other (9.9%).

#### 3.1.4. Prevalence of Mystical Experiences

**Figure 2:**
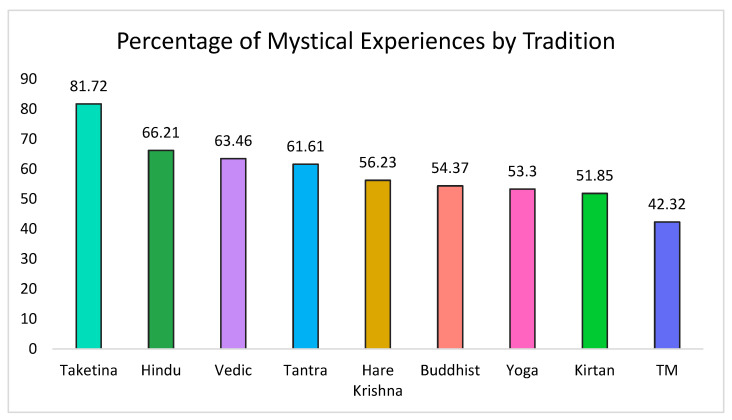
Proportion of participants in each tradition who reported a complete mystical experience.

A total of 60% of participants scored over 60% on all factors of the MEQ30 and therefore met the criterion for a complete mystical experience associated with their chanting practice [56,58]. Figure 2 displays the percentage of participants within the main reported traditions who reported having a complete mystical experience.

### 3.2. Inferential Statistics

#### 3.2.1. Mystical Experience

Three independent-sample t-tests were conducted to determine whether absorption, altruism, and religiosity scores differed between individuals who met the criterion for a mystical experience, compared to those that did not. Levene’s test indicated unequal variances for religiosity, so degrees of freedom were adjusted from 462 to 327. Participants who experienced complete mystical states were higher on absorption scores (*M* = 88.20, *SD* = 19.47) than participants who did not report these experiences (*M* = 67.48, *SD* = 20.52; *t*(462) = 10.910, *p* < 0.0001, *d =* 1.03). Participants who experienced complete mystical states were higher on altruism scores (*M* = 55.23, *SD* = 7.72) than participants who did not report these experiences (*M* = 52.40, *SD* = 7.68; *t*(462) = 3.889 *p* < 0.0001, *d =* 0.37). Participants who experienced complete mystical states were higher on religiosity scores (*M* = 79.24, *SD* = 17.91) than participants who did not report these experiences (*M* = 70.97, *SD* = 13.63; *t*(327) = 5.395, *p* < 0.0001, *d =* 0.52).

A multiple regression was used to establish whether absorption, altruism and religiosity predicted mystical experience scores as measured by the MEQ30. All three predictors accounted for a significant unique percentage of variance in mystical experience, with the overall model explaining 35.4% of the variance in mystical experience scores, *F*(3460) = 83.946, *p* < 0.001. Regression coefficients, confidence intervals, and standard errors can be found in Table 1.

#### 3.2.2. Mystical Experience and Type of Chanting

A two-way ANOVA was used to determine whether mystical experience scores differed between vocalization (vocal or silent), and social context (group or individual) chanting preferences. The ANOVA revealed no effect of vocalization, *F*(1463) = 0.222, *p* = 0.637, *η_p_^2^* = 0.000, and no effect of social context, *F*(1463) = 0.384, *p* = 0.536, *η_p_^2^* = 0.001. There was also no interaction of vocalization and social context on mystical experience, *F*(1463) = 0.245, *p* = 0.621, *η_p_^2^* = 0.001.

#### 3.2.3. Mystical Experience and Traditions

A chi square test for association was conducted between chanting traditions (Vedic chanting, Hindu, Buddhist, Yoga, Hare Krishna, Taketina, Tantra, TM, and Kirtan) and complete mystical experience (yes or no). All expected cell frequencies were greater than five. There was no significant association between mystical experience and tradition, χ^2^ (8) = 12.573, *p* = 0.127.

**Figure 3:**
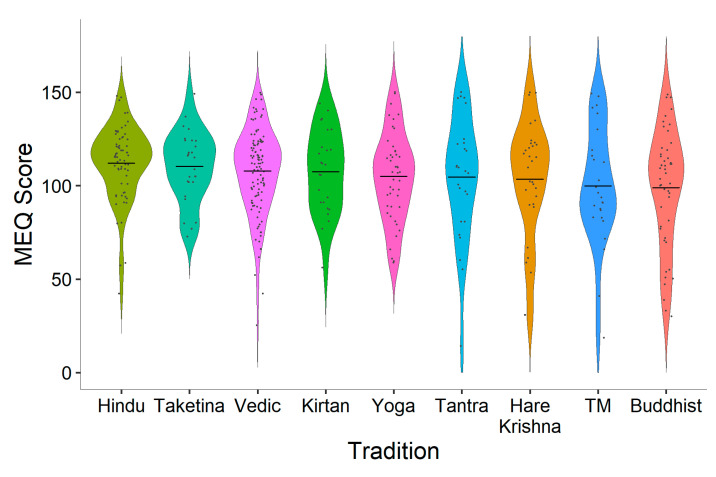
Total mystical experience scores by tradition.

A one-way ANOVA was used (excluding participants in the ‘other’ category) to determine whether mean mystical experience scores differed across traditions. The ANOVA revealed no main effect, *F*(8392) = 1.367, *p* = 0.209. Although there was no significant difference found between chanting traditions’ mystical experience scores, Figure 3 shows that there was a high variability of total mystical experience scores for most traditions, and Hindu participants had the highest scores overall. Table 2 reports the means and standard deviations for the four subscales of the mystical experience questionnaire. 

A two-way mixed ANOVA (excluding participants in the ‘other’ category) was used to determine whether mean scores differed on the four mystical experience questionnaire subscales as a function of tradition. The ANOVA revealed a main effect of subscales, *F*(3,1176) = 85.870, *p* < 0.0001, *η_p_^2^* = 0.180. Chanting leads to high ratings of positive mood (*M* = 3.773, *SD* = 0.045) and ineffability (*M* = 3.765, *SD* = 0.054), followed by mysticism (*M* = 3.470, *SD* = 0.052), and then finally transcendence (*M* = 3.248, *SD* = 0.056).

The ANOVA also revealed an interaction between mystical experience subscale scores and tradition, *F*(24,1176) = 1.828, *p* = 0.009, *η_p_^2^* = 0.036. For mysticism, the tradition that scored the highest was Hindu, whereas TM was lowest on mysticism. For positive mood, the traditions associated with the highest scores included Hare Krishna, Hindu and Kirtan. For ineffability and transcendence subscales, Taketina had the highest scores while Buddhism and TM were comparatively low on ineffability and Hare Krishna was relatively low on transcendence.

## 4. Discussion

The current study set out to shed light on the prevalence and nature of mystical experiences across chanting traditions. In this study, 60% of participants were found to have a ‘complete mystical experience’ during chanting. It should be acknowledged that recruitment by self-selection could give rise to bias, with higher participation rates by individuals who have profound experiences than by individuals who do not. However, the incidence of mystical states observed in our investigation is compatible with the incidence reported in previous research on psychedelics and meditation [14,27,60]. As predicted, participants high in absorption, altruism and religiosity were more likely to have a mystical experience, compared with participants low on these traits. Unexpectedly, mystical experiences were not induced more often by vocal and group chanting than by silent or individual chanting. Also contrary to predictions, chanting traditions with strong beliefs or complex rhythmic patterns were not more likely than other traditions to give rise to mystical states. However, there were significant differences found across the mystical experience subscale scores, suggesting nuanced variations in mystical experiences or interpretations in different chanting traditions. The current findings inform a framework for understanding the universality of chanting practices and the potential of chanting to lead to mystical states.

Having a mystical experience is frequently reported as being among the most meaningful events in a person’s life, and these occurrences are often associated with positive outcomes such as prosocial behaviour, increased self-awareness, health and well-being [4,17,61]. The consistently high percentage of participants reporting mystical experiences across traditions suggests that chanting might induce mystical experiences through features that are common in most traditions, such as focused attention, repetition, rhythm, synchrony, and belief. This interpretation aligns with Stace’s [13] characterisation of the universality of mystical experiences, which claims that all mystical experiences have a common core of phenomenological features, independent from the interpretation of the experiences. In this view, even mystical experiences triggered by psychoactive substances should have a subjective phenomenology and persisting transformative effects that are indistinguishable from experiences that occur in religious contexts.

### 4.1. Traits Influencing Mystical Experience

We found evidence that levels of absorption, altruism, and religiosity distinguished participants who did and did not experience mystical states. Absorption is associated with abilities to focus attention on a specific object and monitor one’s internal state [49,62]. This capacity for heightened internal focus and increased attention may enhance the effects of chanting, making it more likely that an individual will have profoundly moving, or even mystical experiences. The finding of higher absorption amongst chanters who had mystical experiences aligns with previous research that has found absorption to be related to self-regulation, hypnotic susceptibility, meditation, and responsiveness to psychedelics [52,53,54]. It appears that individuals higher in absorption are more predisposed to experiencing altered states in general, and this may manifest as greater rates of mystical experiences in the context of chanting [50].

Altruism was higher among participants reporting mystical experiences. This could be because increased interpersonal skills, particularly when accompanied by rhythmic stimuli, encourages synchronization. For example, children with higher social capacities were found to synchronize better in a dyadic drumming task than children low on social skills [63]. Similarly, high empathy has been linked to rhythmic coordination with people high on trait empathy found to entrain movements quicker to a rhythmic stimulus than those low on trait empathy [64]. Enhanced sensitivity for synchronous aspects of chanting practice is likely to facilitate the experience of altered states by increasing the salience and intensity of repetitive rhythmic stimuli [28].

Finally, individuals who reported mystical states were higher in religiosity, highlighting the role of intentions and beliefs in generating mystical experiences. Previous research has shown that individuals’ beliefs and expectations can influence the degree to which they experience altered states in the contexts of hypnosis [65], religious rituals [66], and group sessions where participants believe they have taken psychedelics [67]. Additionally, a survey comparing naturally occurring mystical experiences to those occasioned by psychoactive substances found that more than two-thirds of participants who identified as atheist prior to a mystical experience no longer identified as atheist afterwards [14]. This change in belief systems suggests the relationship between mystical experience and religiosity may go both ways, with people perhaps becoming higher on religiosity after having such experiences. The current findings suggest that beliefs and expectations may have a role in generating alterations of consciousness in the context of chanting or that mystical experiences may have a role in generating belief systems.

### 4.2. Traditions and Mystical Experience

Taketina, one of the more rhythmically challenging and highly synchronized group chanting practices, had the highest proportion of participants reporting mystical experiences. Further, Taketina practitioners consistently scored in the higher range of the mystical experience questionnaire whereas most of the other traditions had greater variation in mystical experience scores. This is surprising as Taketina is not associated with specific belief systems. We must conclude from this that although particular beliefs may facilitate altered states, they are not a necessary requirement (at least in the context of chanting). There are likely multiple pathways to experience a mystical state. For example, such states might be achieved through complex rhythmic chanting in groups, or through devotional practices. In one case, it may be rhythm that is the primary trigger for the altered state, and in the other, it may be meaning, devotion or surrender associated with a strong belief system. Taketina requires participants to incorporate three contrasting rhythms. This complex polyrhythm not only promotes full-body engagement with clapping, stepping, and chanting, but creates novel, unpredictable emergent patterns that encourage sustained attention. These variable rhythmic structures are more likely to promote concentration in comparison to predictable rhythms that, once known, are no longer salient stimuli [68]. Therefore, it may be both the musical capacity and belief systems that contribute to mystical experiences either individually or together.

### 4.3. Mystical Subscales

Although there was no significant difference between chanting traditions on the total mystical experience score, there were some differences found across the subscales of the mystical experience questionnaire: mystical, positive mood, transcendence of time and space, and ineffability. The mysticism subscale is related to feelings of oneness, fusion of personal self into a larger whole, and experiencing something profoundly sacred and holy. Hindu chanting had the highest scores on this subscale. This could be because Hindu chanting has a relatively esoteric belief system and a strong ceremonial component. Meaningful rituals can promote feelings of transcendence and are enhanced through repetition of the ritual [25,69]. By contrast, TM and Buddhist practices were low on the mysticism subscale. TM is the repetition of a sound or phrase while disregarding everyday thoughts and does not encourage belief systems of merging with something greater [70,71,72] while Buddhist practices are often focused on maintaining awareness of bodily sensations and thoughts [73,74]. TM and Buddhist practices may also require more discipline and attention skills, being practices that are performed mostly alone and silently. Therefore, these differences in ritual and beliefs could be the resulting difference in experiences of oneness or merging with a larger whole.

Positive mood in mystical experiences includes a range of emotions such as ecstasy, joy, peace, and tranquility. Hare Krishna, Hindu and Kirtan traditions had the highest scores on the positive mood subscale. These chanting practices promote a conversational style of chanting that is not only socially interactive but can also become quite ecstatic. This call and response style of chanting could provide a stronger sense of interaction promoting perception–action coupling, the coordination between visual cues and movement [75]. This social interaction, in turn may promote positive mood. Research has found that actions promote stronger responses in an observer if the movement is interactive rather than imitated [41]. Furthermore, group music interactions can increase levels of neurohormones such as dopamine (pleasure and reward), serotonin (mood regulation) and oxytocin (social bonding) also likely leading to increased positive mood [47,76]. Conversely, other practices that scored lower on the positive mood scale, such as TM and Buddhist practices, are practices that are often performed alone with no movement or external music [77,78,79]. Therefore, these practices are not as highly interactive and may not give rise to the same social aspects that may enhance positive mood.

Transcendence involves a loss of usual sense of time and space, which can evolve through repetition or complex rhythm involving high levels of physical and mental engagement [68,80]. Taketina was considerably higher on the transcendence subscale whereas Hare Krishna and Buddhist traditions were relatively low. These latter practices are not rhythmically complex. Although Hare Krishna practices involve high levels of participation with clapping and stepping, movements are not choreographed and the rhythms are more simplistic, making it less cognitively demanding. Csikszentmihalyi found that intense focus on activities, such as jazz improvisation in expert musicians, inhibited self-referential processing, and was related to states of flow in which an individual was fully immersed in a task, often forgetting about themselves, others and the world around them [4]. Furthermore, studies have found that self-referential processes are driven by the posterior cingulate cortex, an area of the brain found to decrease under the influence of psychedelic drugs and mindfulness meditation practices [17]. Taken together, these findings suggest loss of time and space, as experienced in transcendence, may be mediated through complex rhythmic entrainment deactivating self-referential thought processes.

Ineffability refers to a sense that profound experiences cannot be described in words. Taketina and Hindu traditions had the highest ineffability scores. Both of these are relatively complex chanting practices. Taketina involves intricate rhythms and physical entrainment. Hindu chanting involves detailed and multifaceted ceremonial and ritual elements. These factors (complex rhythms and complex ritualistic elements) demand considerable levels of attention and cognitive effort from chanting participants. This high level of cognitive capture likely reduces the capacity for additional mental processes and may lead to reduced levels of self-referential thought. For example, embodied experiences of engaging in rigid, sequenced, repetitive actions requiring motor control make practices more attention grabbing and memorable than non-ritualized practices [25]. Consequently, participants in these practices likely experience reduced attention toward narratives involving self-experience, which may account for the difficulties describing these experiences.

By contrast, Transcendental Meditation (TM) and Buddhist chanting scored quite low on ineffability. These practices are comparatively straightforward, with well-defined and highly repetitive behaviours required of participants. Additionally, these practices explicitly cultivate high levels of precise concentration on one’s internal mental experience. Consequently, practitioners appear more able to describe their subjective experiences. It appears that practices such as TM and Buddhist chanting may trigger mystical states due to deeply held belief systems or practical attention skills that allow practitioners to transcend mundane awareness without engaging in significant physical activity. On the other hand, Taketina and Hindu traditions are often vocal, including full-body engagement and multitasking which could assist in maintaining focus whether attentional skills and beliefs are present or not [81]. Mystical states with high levels of embodiment may be more difficult to describe than those with relatively less embodiment.

### 4.4. How Does Chanting Promote Mystical States?

**Figure 4:**
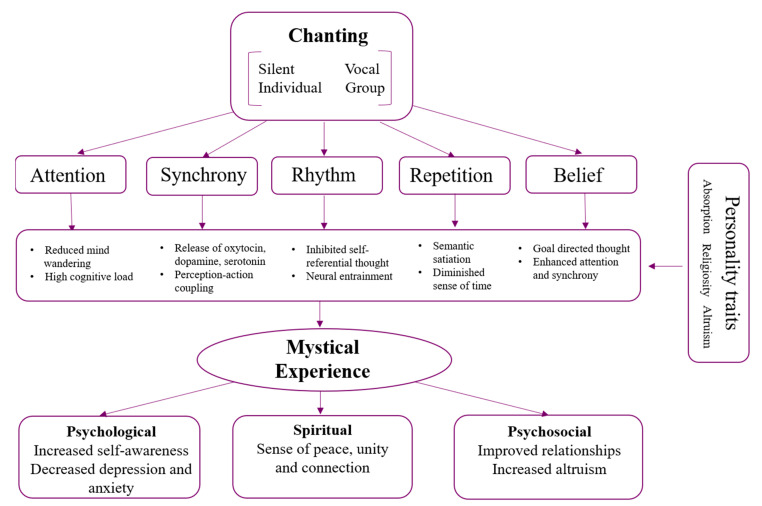
Model of chanting features, mechanisms, traits, and benefits that may arise from mystical states.

We propose a model of how chanting may contribute to mystical states, based on previous empirical and theoretical research of music, meditation, and psychedelics. Although components of the model were not directly manipulated in this study, we have based the model on common features of chanting practices. Figure 4 illustrates five core features of chanting; attention, repetition, synchrony, rhythm, and belief, which can lead to changes across a range of neurocognitive mechanisms giving rise to mystical experiences. These mystical states, in turn, may lead to positive psychological, spiritual, and psychosocial outcomes such as decreased anxiety and depression, and increased social connection.

Capacities of chanting (attention, repetition, synchrony, rhythm, and belief) work both individually and together, leading to neurocognitive functions that are associated with mystical states. Attention and repetition promote disengagement from automatic thoughts, reduced mind wandering, and can diminish a sense of time through semantic satiation, when a phrase temporarily loses its meaning [68,82,83]. Rhythm and synchrony promote perception–action coupling and neural entrainment, as well as inhibit self-referential thought [41,84]. Further, synchronous music activities lead to neurohormonal changes, such as increased dopamine, serotonin and oxytocin [47,48]. The release of these hormones also plays an important role in psychoactive induced altered states and, in the trait, absorption [50]. Lastly, traits we found to be positively associated with mystical states may enhance some of the capacities of chanting in our model. For example, absorption could promote higher levels of attention; altruism may encourage synchrony and rhythm; and religiosity may enhance both attention and synchrony.

A number of questions remain unanswered, which may guide the direction of future research. First, the correlational nature of this research makes it difficult to infer causation. For example, it is unclear whether someone high in certain traits is more likely to have mystical experiences, or if such traits are enhanced through mystical experiences. Such questions of causation might be clarified in longitudinal studies that include repeated measures on various traits, to assess whether they are changed by long-term chanting practice. Second, our research focused on positive mystical experiences as measured by the MEQ30, but not all mystical states are positive. Thus, it may be useful to investigate unhealthy or pathological mystical experiences during chanting, and the possible connection between psychopathology and mystical states. Individuals prone to psychosis are not more likely to experience mystical states, but there are intriguing points of overlap between mystical states and states of psychosis, delusion and schizophrenia [4,85]. For example, paranormal beliefs have been found to be associated with likelihood of experiencing mystical states [86]. Finally, it may be valuable to consider a wider range of altered states than the mystical experiences examined here in order to provide greater insight into the mechanisms by which chanting gives rise to such states.

## 5. Conclusions

The current findings provide a framework for understanding the universality of chanting practices and the potential of chanting to lead to mystical states. This extends previous research on altered states, contemplative practices and psychedelics [14,27,59]. We found that practitioners of many styles of chanting reported mystical experiences to varying degrees and identified three individual difference characteristics—absorption, altruism, and religiosity—that were associated with greater levels of mystical experiences in the context of chanting. Whether other traits predict mystical and other states of consciousness, including positive and negative states, remains an important question for future research. Our findings reveal that chanting, whether practiced silently, vocally, individually or in a group, is a reliable method of inducing profound altered states of consciousness. Such states, and the capacity of chanting to achieve them, may have beneficial applications for physical and mental health, and provide a deeper understanding of spiritual beliefs and practices that are an integral part of human culture.

## Figures and Tables

**Table 1:** Regression results for mystical experience scores.

	*B*	95% CI for *B*		*SE B*	*t*	*p*
		LL	UL			
Constant	18.801	3.719	33.883	7.675	2.45	0.015
Absorption	0.542 **	0.449	0.636	0.048	11.35	0.000
Altruism	0.353 *	0.091	0.614	0.133	2.65	0.008
Religiosity	0.320 **	0.191	0.449	0.066	4.88	0.000

Note: *B* = unstandardized regression coefficient; CI = confidence interval; LL = lower limit; UL = upper limit; *SE B* = standard error of the coefficient. * *p* < 0.01 ** *p* < 0.001.

**Table 2:** Revised mystical experience questionnaire (MEQ30) subscale mean and standard deviation (in parentheses) scores by tradition.

Subscale of MEQ30	Hindu	Taketina	Vedic Chanting	Kirtan	Yoga	Tantra	Hare Krishna	TM	Buddhist
Mystical	3.76 (0.68)	3.54 (0.73)	3.60 (0.85)	3.51 (0.90)	3.44 (0.93)	3.45 (1.19)	3.40 (1.07)	3.24 (1.22)	3.30 (1.10)
Positive mood	3.97 (0.63)	3.85 (0.65)	3.79 (0.74)	3.98 (0.58)	3.76 (0.70)	3.64 (1.10)	3.88 (0.92)	3.59 (1.02)	3.50 (1.01)
Transcendence	3.35 (0.94)	3.67 (0.74)	3.26 (0.89)	3.21 (0.87)	3.17 (0.92)	3.35 (1.13)	2.97 (1.31)	3.21 (1.08)	3.04 (1.22)
Ineffability	3.91 (0.97)	4.04 (0.63)	3.82 (0.84)	3.89 (0.83)	3.89 (0.78)	3.65 (1.13)	3.74 (1.14)	3.47 (1.02)	3.50 (1.30)

## Data Availability

The data presented in this study are openly available in [OSF] at doi:10.17605/OSF.IO/VFY8C.
